# 658. Assessment of Initial Burn Care Knowledge Amongst Emergency Medicine Personnel

**DOI:** 10.1093/jbcr/irag033.495

**Published:** 2026-04-06

**Authors:** Savannah Carroll, Bailey Segura, Debra Philpot, Tommy Tran, Jorge Vega

**Affiliations:** TriStar Skyline Medical Center, Nashville, TN; TriStar Skyline Medical Center, Nashville, TN; TriStar Skyline Medical Center, Nashville, TN; TriStar Skyline Medical Center, Nashville, TN; TriStar Skyline Medical Center, Nashville, TN

## Abstract

**Introduction:**

Proper initial burn care by EMS providers is critical for improving patient outcomes and minimizing morbidity and mortality, particularly in the immediate post-injury period. Despite its significance, studies show variations exist in prehospital burn care, from fluid resuscitation protocols to pain management and wound dressing, which can lead to suboptimal patient care. Furthermore, a lack of regular, burn-specific education contributes to low EMS provider confidence and limited knowledge of current evidence-based practices. By characterizing and evaluating current knowledge and practices of prehospital providers regarding initial burn care, this study sought to identify specific areas for educational improvement and inform the development of targeted training programs.

**Methods:**

A 10-question survey assessing initial stabilization and management, fluid resuscitation, burn depth and size, and guidelines for burn patient referral was distributed during EMS courses, through social media platforms, in EMS lounges, and were sent to EMS leadership. Respondent demographics were obtained utilizing 9 survey questions. Survey design, access (QR code), and initial analysis were completed utilizing Microsoft Forms.

**Results:**

A total of 163 survey responses were received over a period of 77 days. Respondents were primarily AEMT (31%) and paramedics (34%). Of those, 21 (13%) were ABLS certified and 88 (54%) indicated moderate confidence in providing initial burn care. Of note, 43 (26%) respondents correctly answered questions regarding initial fluid resuscitation rates and proper TBSA calculation. Further, 86 (53%) respondents correctly identified the most reliable indicator of inhalation injury. Further analysis suggests an association between initial fluid resuscitation rate choices, EMS certification level, and ABLS certification. Similar associations are noted with TBSA calculation responses and respondent level of confidence.

**Conclusions:**

This study identified knowledge gaps and variations in prehospital assessment of burn severity and management of burn injuries, specifically related to initial fluid resuscitation rates, proper TBSA calculation, and indicators of inhalation injury. These findings, along with EMS provider level of confidence, were associated with EMS certification level and ABLS training, highlighting a potential correlation between educational background and practice knowledge. This indicates a need for focused, high-impact education to improve provider confidence and bridge the gap between current knowledge and evidence-based standards for initial burn care.

**Applicability of Research to Practice:**

By addressing these educational needs through targeted educational programs, the quality and consistency of prehospital burn care can be standardized and enhanced, thereby improving outcomes for patients with burn injuries.

**Funding for the study:**

N/A.

